# Impact of national-scale targeted point-of-care symptomatic lateral flow testing on trends in COVID-19 infections, hospitalisations and deaths during the second epidemic wave in Austria (REAP3)

**DOI:** 10.1186/s12889-023-15364-w

**Published:** 2023-03-16

**Authors:** Stephanie Reitzinger, Thomas Czypionka, Oliver Lammel, Jasmina Panovska-Griffiths, Werner Leber

**Affiliations:** 1 Institute for Advanced Studies, Vienna, Austria; 2 London School of Economics and Political Science, London, UK; 3 Practice Dr. Lammel, Ramsau am Dachstein, Austria; 4 The Big Data Institute and The Pandemic Sciences Institute, University of Oxford, Oxford, UK; 5 The Queen’s College, University of Oxford, Oxford, UK; 6 Wolfson Institute of Population Health, Barts and The London School of Medicine and Dentistry, Queen Mary University of London, London, UK

**Keywords:** SARS-CoV-2, Symptomatic lateral flow testing, Statistical analysis

## Abstract

**Background:**

In October 2020, amidst the second COVID-19 epidemic wave and before the second-national lockdown, Austria introduced a policy of population-wide point-of-care lateral flow antigen testing (POC-LFT). This study explores the impact of this policy by quantifying the association between trends in POC-LFT-activity with trends in PCR-positivity (as a proxy for symptomatic infection), hospitalisations and deaths related to COVID-19 between October 22 and December 06, 2020.

**Methods:**

We stratified 94 Austrian districts according to POC-LFT-activity (number of POC-LFTs performed per 100,000 inhabitants over the study period), into three population cohorts: (i) *high*(*N* = 24), (ii) *medium*(*N* = 45) and (iii) *low*(*N* = 25). Across the cohorts we a) compared trends in POC-LFT-activity with PCR-positivity, hospital admissions and deaths related to COVD-19; b) compared the epidemic growth rate before and after the epidemic peak; and c) calculated the Pearson correlation coefficients between PCR-positivity with COVID-19 hospitalisations and with COVID -19 related deaths.

**Results:**

The trend in POC-LFT activity was similar to PCR-positivity and hospitalisations trends across *high, medium* and *low* POC-LFT activity cohorts, with association with deaths only present in cohorts with *high* POC-LFT activity. Compared to the *low* POC-LFT-activity cohort, the *high*-activity cohort had steeper pre-peak daily increase in PCR-positivity (2.24 more cases per day, per district and per 100,000 inhabitants; 95% CI: 2.0–2.7; *p* < 0.001) and hospitalisations (0.10; 95% CI: 0.02, 0.18; *p* = 0.014), and 6 days earlier peak of PCR-positivity. The *high*-activity cohort also had steeper daily reduction in the post-peak trend in PCR-positivity (-3.6; 95% CI: -4.8, -2.3; *p* < 0.001) and hospitalisations (-0.2; 95% CI: -0.32, -0.08; *p* = 0.001). PCR-positivity was positively correlated to both hospitalisations and deaths, but with lags of 6 and 14 days respectively.

**Conclusions:**

High POC-LFT-use was associated with increased and earlier case finding during the second Austrian COVID-19 epidemic wave, and early and significant reduction in cases and hospitalisations during the second national lockdown. A national policy promoting symptomatic POC-LFT in primary care, can capture trends in PCR-positivity and hospitalisations. Symptomatic POC-LFT delivered at scale and combined with immediate self-quarantining and contact tracing can thus be a proxy for epidemic status, and hence a useful tool that can replace large-scale PCR testing.

**Supplementary Information:**

The online version contains supplementary material available at 10.1186/s12889-023-15364-w.

## Introduction

**Fig. 1:**
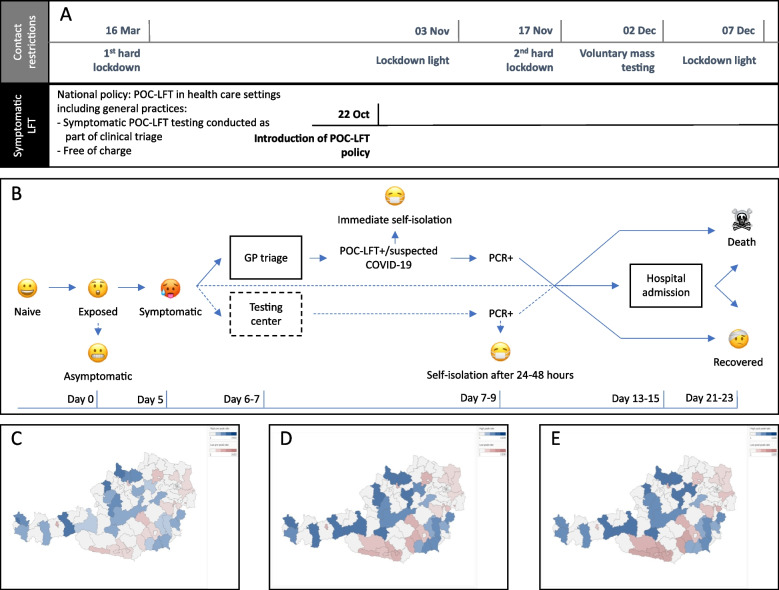
**A-E** Key dates of the Austrian social distancing policy (**A**), the Austrian POC-LFT policy (**B**), and heatmaps of COVID-19 PCR positivity during the study period (**C-D**). **A**: Timeline of the different national social distancing policy restrictions during the first wave and the beginning of the second wave, and details of the Austrian symptomatic point-of-care lateral flow antigen tests (POC-LFT) policy introduced as of October 22, 2020. **B**: Description of the clinical pathway recommended by the Austrian COVID-19 testing policy, including the possible outcomes of SARS-CoV2 infection during the study period. **C-E**: Heatmaps of the PCR positive cases before (**C**), during (**D**) and after (**E**) the second COVID-19 wave between October 22 and December 06, 2020, across the 94 Austrian districts studied. The geographical maps (**C-E**) were generated using the free Tableau Public software (https://public.tableau.com/en-us/s/); districts with *high* POC-LFT use are labelled in blue and shaded according to their activity, and districts with *low* activity are shaded in red. For clarity, districts with *medium* activity (grey) were not shaded. POC-LFT: point-of-care lateral flow antigen test.

In March 2022, at the time of writing and over two years since the first case of COVID-19 was identified, the epidemic continues to spread across the world driven by emerging SARS-CoV-2 variants [1]. As some nations are starting to lift imposed lockdowns and moving towards a “living with COVID-19” endemic state, it is important to retrospectively evaluate strategies that can continue to identify and curb population-wide epidemic growth [2]. For example, our group has previously reported that large-scale point-of-care antigen lateral flow testing (POC-LFT) can be an accurate alternative to SARS-CoV-2 Polymerase Chain Reaction (PCR) testing in primary care when combined with clinical assessment in symptomatic patients in Austria [3]. Our results [3, 4] were used as part of the evidence to inform a nationwide policy to provide and reimburse targeted POC-LFT for COVID-19 in symptomatic patients in primary care [5]. As part of the policy, clinicians used POC-LFT in clinical triage for patients presenting with mild to moderate flu-like symptoms and notified public health authorities of any reactive POC-LFT result on the same day, enabling early self-quarantining and contact tracing. This policy came into effect in October 2020, at a time when the 94 Austrian districts were in different phases of epidemic growth amidst the second national epidemic wave (Fig. 1A). In the study presented in this paper we extend our previous work to evaluate the impact of this policy and specifically explore whether large-scale targeted POC-LFT of symptomatic patients in primary care can capture PCR-positivity, as well as trends in hospitalisations and deaths related COVID-19 during the second epidemic wave in Austria in the autumn of 2020.

Vaccination against COVID-19 infection is an effective tool to reduce the burden of the infections, reduce ongoing transmission within a population and lead to reduced incidence and prevalence of SARS-CoV-2 infections on a large scale [6]. The mass roll-out of a vaccination programme in late 2020 has led to reduced transmission, hospitalisations and death related to COVID-19 across the world [7]. However, the impact has been different across different countries and affected by vaccine efficacy, vaccine hesitancy and scarcity of vaccine allocation across settings [8]. Non-pharmaceutical interventions (NPIs) that complement vaccination programmes remain an important tool for outbreak control. These include use of face coverings, physical distancing measures, travel restrictions [9] and Test-Trace-Isolate strategies (TTI) that allow for a targeted large-scale testing, early isolation of positive cases and tracing of their contacts [10].

Viral detection via population-wide testing is a crucial first step in reducing ongoing transmission via capturing infected people, isolating them and informing and quarantining their contacts [11]. Due to its high accuracy, PCR testing is considered the gold-standard for viral detection [12]. However, PCR tests can be costly, may incur a delayed result reporting, may be subject to lab capacity limitations especially during periods of high demand and under the ‘living with COVID-19’ strategies. During 2022, testing has become more scarce with some countries only using them in hospital settings (e.g. in England from April 01, 2022) [13]. These issues raise concerns about PCR utility during both major possible future outbreaks and as an ongoing method for viral detection.

Lateral flow test devices (LFTs) can be a safe, affordable and accurate alternative to PCR, when used within five days from symptom onset [12]. In high prevalence settings (≥ 5%), the World Health Organisation (WHO) recommends prioritisation of antigen-detection rapid diagnostic tests (Ag-RDTs) such as LFT as a TTI strategy for early symptomatic cases [14]. LFTs can be quickly produced in large quantities, and the results can be obtained on site in 15 to 30 min without the need for a laboratory. A reactive POC-LFT result also allows for immediate self-isolation of suspected cases and timely contact tracing.

This work extends our previous findings on the utility and accuracy of LFTs to explore the impact of an Austrian policy recommending nation-wide targeted symptomatic POC-LFT on key outbreak indicators during the early phases of the second epidemic wave in late 2020 [3]. The aim of the study was to explore this impact by calculating the association between the number of symptomatic people testing POC-LFT reactive with PCR-positive cases, hospitalisations and deaths across all 94 political districts in Austria.

## Methods

### Data

The study is covering a period of three months during the peak of the second COVID-19 epidemic wave, defined as the period between introduction of the Austrian POC-LFT policy on October 22, 2020 [5], and the end of the second hard national lockdown on December 06, 2020 (Fig. 1A). We note that while there are no clear definitions of commonly used adjectives for the terms soft/hard lockdown, using the matrix definition in [15], we define lockdown as a set of measures aimed at reducing transmission of COVID-19 that are mandatory, applied indiscriminately to a general population and involve some restrictions on the established pattern of social and economic life. For the purposes of our study, we define the differences between hard and soft to therefore lie in the number of contacts to be avoided. A soft lockdown aims at reducing the number of contacts but not stopping then, while a hard lockdown aims at minimizing them fully and can therefore be a proxy for full lockdown with most of the societal infrastructure closed.

During the study period, any patients suffering from typical COVID-19 symptoms, and those testing LFT reactive, were categorised as suspected COVID-19 cases and recommended immediate self-isolation (Fig. 1B).

We used three different datasets for each of the 94 Austrian districts for the period between October 01 to December 31, 2020. Firstly, we used the reimbursement data of the largest Austrian health insurance provider (Österreichische Gesundheitskasse, Austrian Health Insurance Fund, https://www.gesundheitskasse.at) covering the monthly sum of reimbursed LFTs per district conducted by contracted general practitioners, and pediatric and internist practices. Through these practices, the Austrian Health Insurance Fund is the sole provider of free symptomatic POC-LFT in the country; and the dataset is a good representation of the level of LFT use across the 94 districts. Secondly, we used the publicly available district-level data of people testing SARS-CoV2 PCR positive and deaths (available at https://covid19-dashboard.ages.at/). The third dataset used were daily COVID-19-related hospital admissions for each district provided by the Austrian National Public Health Institute (Gesundheit Österreich GmbH, https://goeg.at/english). All data on PCR, hospital admissions and deaths were stratified per age group and sex.

### Stratification of districts by POC-LFT-activity

To explore the impact of POC-LFT among symptomatic patients, we stratified the 94 districts according to their POC-LFT-activity (or relative POC-LFT testing rate), defined as the number of POC-LFT performed per 100,000 inhabitants between October 2020 and December 2020 (Figs. 1C-E). Based on this intensity of testing, we split the population into three cohorts with different levels of (POC-LFT) testing: (i) *high*-POC-LFT-activity, (ii) *medium*-POC-LFT-activity and (iii) *low-*POC-LFT-activity (see figure S2 in the supplementary materials).

The process to define these three cohorts was iterative, noting that most (90%) POC-LFTs were conducted by contracted general practitioners, with only a small fraction (10%) performed by contracted pediatricians and internists. To generate the three cohorts, we first identified districts with the highest POC-LFT-activity in October 2020 and took these to represent the “top third” testing level districts. However, rather than using the top third of the 94 districts (i.e. the top 31 districts) we included the top 37 districts to include districts that had very similar levels of POC-LFT testing activity in October 2020. Of these 37 districts in October 2020, districts that remained with high levels of POC-LFT testing until December 2020 were categorised as *high*-POC-LFT-activity districts. Analogously, we searched for the “bottom third” of districts, i.e. those districts with lowest testing POC-LFT levels in October 2020. Districts that remained with low POC-LFT level until December 2020, were categorised as *low*-POC-LFT-activity districts. Finally, districts, which were neither *high*- nor *low*-POC-LFT-activity districts, were categorised as districts with *medium-*POC-LFT-activity.

### Statistical analysis

Across the three population cohorts we undertook three separate analyses. Firstly, we compared trends in POC-LFT-activity by cohort to the prevalence of COVID-19 related infections, hospital admissions related to COVID-19, and deaths related to COVD-19 over the three-month-period. Secondly, we quantified the differences in the slopes of COVID-19 PCR-positivity between *high* and *low* POC-LFT-activity cohorts, during the rise and the decline of PCR-positivity from the (effective) beginning of reimbursed POC-LFT up to the end of the national lockdown (October 25 to December 06) and using multiple-group interrupted time series analysis (ITSA) (Linden & Arbor, 2015). Across 49 observations per day, 25 in the *high* and 24 in the *low* cohort, and over 42 days we used ITSA with the following assumptions a) we consider a lag for effectiveness of three days to account for the fact that the period started with the introduction of reimbursement of POC-LFT by the health insurance fund to the contracted practices; b) we divided the period as pre-peak and post-peak of infections noting that COVID-19 infections started to decline between the enforcements of the closing of the gastronomy on November 03 (lockdown light) and the second hard national lockdown on November 17; c) the period ended with the last day of the hard national lockdown on December 06 (Fig. 1A).

To quantify the difference in the growth rate across cohorts, we fitted an Ordinary Least Squares (OLS) statistical model and tested for autocorrelation of the error distribution with the actest proposed by Cumby and Huizinga [16]. The model included two time lags and had the following form:

1 $${Y}_{t}={\beta }_{0}+{\beta }_{1}{T}_{t}+{\beta }_{2}{high}_{t}+ {\beta }_{3}{high}_{t}{T}_{t}+ {\beta }_{4}post+ {\beta }_{5}post{high}_{t}+{\beta }_{6}post{T}_{t} +{\beta }_{7}post{{high}_{t}T}_{t}+{\varepsilon }_{t}$$

The dependent variable Y was the weekly rolling average of PCR positive cases or weekly rolling average of hospital admissions across different district cohorts. We identified the *high* district cohort with the dummy variable *high* and the time since the start of the study (October 22) with T (and t each time point). We indicated the days after the peak of COVID-19 PCR-positivity or hospital admissions with the dummy variable *post*. The coefficients of interest were the interaction of *high* and t (*high_t*), indicating the difference in slopes before the peak, and, the interaction of post, *high* and t (*post_high_t*), indicating the difference in slopes after the peak between the cohorts with *high* and *low* POC-LFT-activity.

Finally, we calculated the Pearson correlation coefficients between PCR-positivity and COVID-19 hospitalisations and between PCR-positivity and COVID -19 related deaths across the *high*, *medium*, and *low* POC-LFT-activity cohorts. We accounted for lags between PCR-positivity and hospitalisation due to COVID-19 and PCR-positivity and COVID-19 related deaths and determined the lag for which the Pearson correlation was strongest.

## Results

Twenty five of the 94 districts were classified as *high* POC-LFT-activity (or *high* cohort), 45 as *medium* POC-LFT-activity (or *medium* cohort*)* and 24 as *low* POC-LFT-activity (or *low* cohort). In total, the *high* and *medium* cohorts covered 1.9 million and 3.4 million people, respectively, while the *low* cohort covered 3.7 million people (including 1.9 million people of the capital Vienna). The median population size per district was 79,593 in the *high*, 60,936 in the *medium* and 64,078 in the *low* cohort. Age and gender of patients testing PCR positive were comparable among all three groups (Table 1). However, patients admitted to hospital were younger in the *low* cohort, compared to the *high* and *medium* cohorts. The number of men admitted to hospital was similar to the number of women admitted to hospital across all three cohorts (Table 1).

**Table 1:** Descriptive statistics for the three study cohorts

	***Cohorts with high POCLFT use***		***Cohorts with medium POCLFT use***		***Cohorts with low POCLFT use***	
Districts (*N* =)	25		45		24	
Total population (*N* =)	1,869,845		3,402,368		3,660,451	
Median (sd) population per district	79,592		60,936		64,078	
Age	PCR^a^ positive	hospital admissions	PCR^a^ positive	hospital admissions	PCR^a^ positive	hospital admissions
0–19	12.2%	1.1%	11.8%	1.1%	11.6%	1.3%
20–49	47.8%	7.0%	47.1%	7.9%	46.1%	9.8%
50–64	23.1%	18.9%	23.3%	18.3%	24.8%	18.0%
65–79	9.8%	33.0%	10.1%	33.3%	10.3%	31.9%
80 +	7.1%	40.0%	7.7%	39.4%	7.2%	39.1%
men	48.3%	54.2%	48.8%	52.1%	48.2%	51.2%
women	51.7%	45.8%	52.2%	47.9%	51.8%	48.8%

^a^*PCR* polymerase chain reaction

**Fig. 2:**
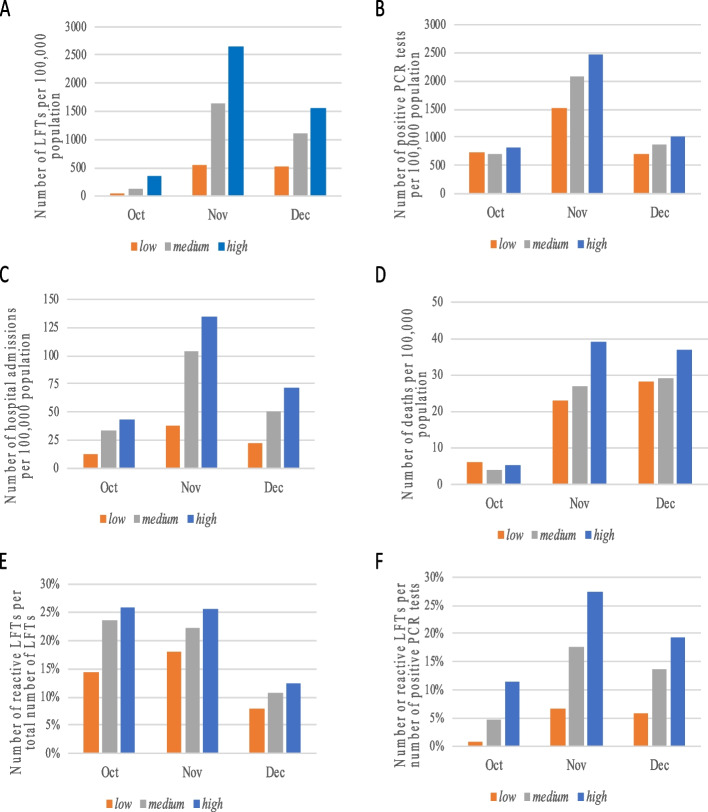
A-F: Comparison of six indicators between study cohorts with *high*, *medium* and *low* POC-LFT. **A**: Monthly ratio (number of people/100,000 inhabitants) of the sum of combined reactive and non-reactive POC-LFT. **B**: Monthly ratio (number of people/100,000 inhabitants) of people with a positive PCR test result. **C**: Monthly ratio (number of people/100,000 inhabitants) of hospital admissions (or hospitalizations) related to COVID-19. **D**: Monthly ratio (number of people/100,000 inhabitants) of deaths related to COVID-19 from October 01 to December 31, 2020, for each of the high, medium and low POCLFT district cohorts. **E**: Monthly proportions of people with reactive POCLFT result to the total number of POC-LFTs received per POC-LFT cohort. **F**: Monthly proportions of people with reactive POC-LFT result to the total number of patients testing PCR positive per POC-LFT cohort. POC-LFT: point-of-care lateral flow antigen test

We found that targeted symptomatic POC-LFT trends agreed with the trends in PCR-positivity and hospitalisations related to COVID-19 across all POC-LFT activity cohorts (Fig. 2A-F). However, the trend in POC-LFT was similar to the trend in deaths only in the cohorts with high POC-LFT activity (Fig. 2A-F). Specifically, there was an increase from October to November and a decline from November to December in the number of POC-LFTs per 100,000 people, PCR-positivity and hospitalisations related to COVID-19. During October and in November symptomatic POC-LFT, PCR-positivity, reactive POC-LFT number, hospitalisations and deaths related to COVID-19 were higher in the *high* POC-LFT-activity cohort (Fig. 2A-F), suggesting that the epidemic had started earlier and was more widespread in the settings with *high* POC-LFT-activity compared to the *medium* and *low* cohorts.

The increase of PCR positivity from October to November and the decline of PCR positivity from November to December was slightly higher in the *high* POC-LFT cohort compared to the *medium* cohort (4.9% greater increase and 2.2% greater decrease in PCR-positivity, and similar increase and 7.9% greater decrease in hospitalisations in Fig. 2A-F). This difference in PCR positivity was much higher in the *high* POC-LFT cohort compared to the *low* cohort (48.8% greater increase and 11.2% greater decrease in PCR-positivity, and similar increase and 7.9% greater decrease in hospitalisations in Figs. 2A-F). The trend in deaths related to COVID-19 was aligned to the trend in the symptomatic POC-LFT testing in the *high* POC-LFT-activity cohort – with an increase from 5 to 39 deaths per 100,000 inhabitants from October to November and decline from 39 to 38 deaths per 100,000 inhabitants from November to December, but the latter was not captured in the *medium* and the *low* cohorts (Fig. 2D). In fact, in the *medium* and *low* POC-LFT-activity cohorts, there was an increase of 7% and 22% respectively, in deaths related to COVID-19 between November and December unlike the rest of the trends (Fig. 2D). Finally, we found that in the *low* POC-LFT-activity cohort the share of reactive POC-LFTs from October to November increased by 27%, while in the cohorts with *medium* and *high* POC-LFT-activity, these remained almost constantly high (Fig. 2E). From November to December the share of reactive POC-LFT decreased similarly across all three cohorts (52% in the cohorts with *high* and *medium* POC-LFT-activity and 56% in the cohort with *low*-activity in Fig. 2E).

Comparing the difference in the slope of PCR-positivity and hospitalisations between the *high* and *low* POC-LFT-activity cohorts (Table 2), we found that compared to the *low* cohort, the *high* cohort had significantly (*p* < 0.001) steeper pre-peak daily increase in PCR-positivity (on average 2.35 vs. 4.59 cases per day, per district and per 100,000 inhabitants) and hospitalisations (on average 0.12 vs. 0.22 cases per day, per district and per 100,000 inhabitants), and an earlier peak of PCR-positivity (6 days earlier). Compared to the *low* cohort, during the second hard national lockdown, the *high* cohort showed a significantly (*p* < 0.001) steeper daily reduction of the pre-peak trend in PCR-positivity (on average 3.6 cases, per day, per district, per 100,000 inhabitants) and hospitalisations (on average 0.2 cases, per day, per district, per 100.000 inhabitants) (see Fig. S1). Hence the rise in symptomatic infections and hospitalisations related to COVID-19 were more pronounced in the cohort with higher POC-LFT-activity (see Table 2), suggesting that POC-LFT-activity in the Austrian setting at that time was illustrative of an epidemic outbreak. Moreover, we observe a steeper decline in PCR-positivity and hospital admissions after the peak in the cohort with the higher POC-LFT-activity, again confirming the trend in symptomatic LFT is aligned with the epidemic metrics’ trends.

**Table 2:** Results including the coefficients and 95% Confidence Intervals (CIs) of the interrupted time series analyses

	***Parameters*** ***of*** ***PCR***^b^ ***positivity*** ***model*** ***model*** ***(***${\varvec{\beta}}$*, ****95%CI)***		***Parameters*** ***of*** ***hospitalizations*** ***model*** ***model*** ***(***${\varvec{\beta}}$, ***95%CI)***	
*high*^c^	8.5	[0.64; 16.3]	0.53	[-0.26; 1.33]
*t*^d^	2.35^***^	[2.0; 2.7]	0.12^***^	[0.07; 0.17]
***high_t***	2.24^***^	[1.4; 3.1]	0.10^+^	[0.02; 0.18]
*post*^e^	88.3^***^	[68.4; 108.2]	3.06^**^	[1.05; 5.08]
*post_high*	52.0^**^	[21.9; 82.1]	4.02^**^	[1.13; 6.9]
*post_t*	-4.03^***^	[-4.7; -3.3]	-0.15^***^	[-0.23; -0.07]
***post_high_t***	-3.60^***^	[-4.8; -2.3]	-0.20^**^	[-0.32; -0.08]
*c*	20.05^***^	[15.9; 24.2]	1.03^***^	[0.47; 1.58]

^***^p < 0.001

^**^p < 0.05

^*^p < 0.10

^+^*p* < 0.15

^a^Coefficients [and 95% CI] of the interrupted time series analyses: The dependent variable is the weekly rolling average of polymerase chain reaction (PCR)

^b^positive cases (2^nd^ column) and the weekly rolling average of hospital admissions (3^rd^ column) across districts with different point-of-care lateral flow antigen tests (POCLFT) use

^c^*high*: dummy variable indicating districts with high point-of-care lateral flow antigen tests (POCLFT) use

^d^*t*: time variable (days)

^e^*post*: dummy variable indicating the time period (in days) after the peak of the 2^nd^ epidemic wave

**Fig. 3:**
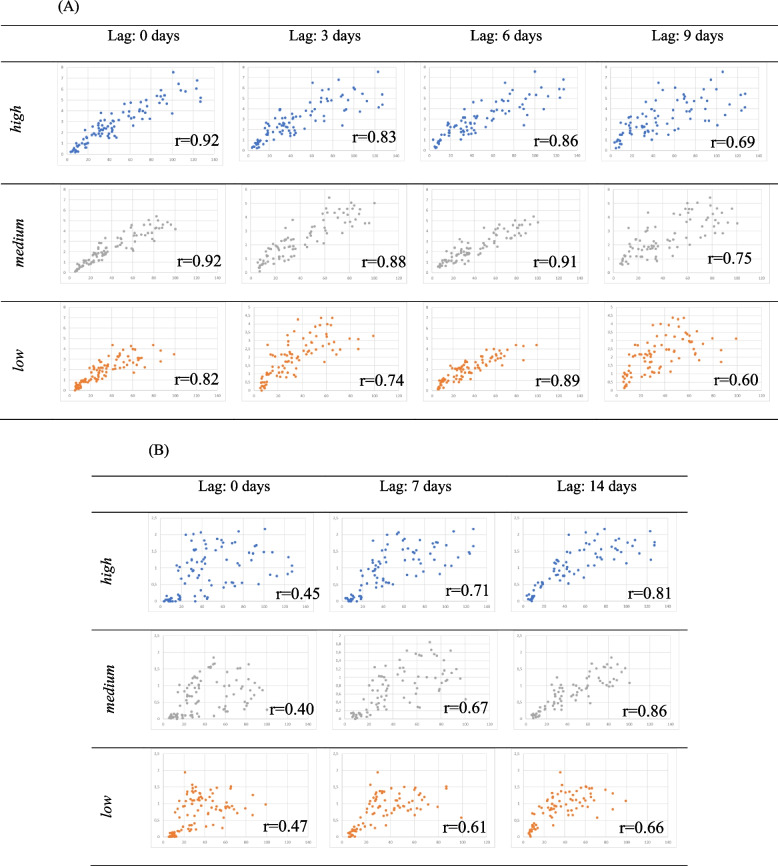
Correlation between PCR-positivity and hospitalisation (**A**), and PCR-positivity and deaths (B), by POC-LFT activity (high, medium, low). The mean of-PCR-positivity is plotted against the x-axis, and the means of hospitalizations and deaths respectively are plotted against the y-axis

Finally, we found a strong correlation between PCR-positivity and hospitalisations with a lag of 6 days, which was similar across all three cohorts (see Fig. 3A-B). We note that in districts with *low* POC-LFT-activity, the correlation seemed to increase marginally with the lag, with 6 days being the lag for optimal correlation. The correlation between symptomatic infections (proxied by PCR-positivity) and COVID-19 related deaths was strongest when considering a lag of 14 days, noting that the correlation was less pronounced in the cohort with *low* POC-LFT-activity.

## Discussion

Data on the impact of population-wide targeted symptomatic POC-LFT in capturing COVID-19 epidemic trends in infections and hospitalisation trends remains scarce. This study shows that implementation of a national policy recommending targeted POC-LFT for people presenting with mild to moderate flu like symptoms to primary care settings, can lead to an increased COVID-19 case finding during a major COVID-19 outbreak, and earlier and statistically significant reduction in cases and hospitalisations during the Austrian second national lockdown. This suggests that increased POC-LFT activity can be an indicator of increased COVID-19 cases and hospitalisations. We did not observe an alignment between symptomatic POC-LFT trends and the trends in deaths related to COVID-19, which could be due to delay in reporting deaths. In fact, we confirm a strong correlation between symptomatic COVID-19 infections (proxied by PCR-positivity) with hospitalisations and deaths from COVID-19 with an average delay of six days between cases and hospitalisations, and 14 days between cases and deaths respectively. This correlation was more robust in settings with *high* POC-LFT-activity suggesting that epidemic trends are more aligned when POC-LFT activity is high, i.e., during an increasing COVID-19 outbreak. We note that that in low and medium cohorts, the death rate still increased, opposite to the trend in the high cohort. We note that while our results indicate this, further data and analysis are needed to distinguish between test activity and epidemic outbreak to fully test whether increased POC-LFT is a proxy for epidemic growth. Furthermore, the delay of 14 days between correlated deaths and cases in a study period of 90 days maybe the reason why we did not see the trend in cases being translated into the trend in deaths. More data on deaths related to COVID-19 over a longer time period is required to explore this further, but this is beyond the scope of this work.

Due to their ability to detect infectious cases early, LFTs have been considered instrumental in early COVID-19 outbreak containment [17]. Population-wide LFT screening combined with immediate TTI in Slovakia suggested association with reduction in COVID-19 infection prevalence [18]. However, this national programme, which was conducted by medical workers and planned as a repeated intervention to prevent a national lockdown, was terminated prematurely, due to lack of material and human resources, and failure to reduce PCR-positivity rates [19]. Hence, indiscriminate whole population LFT screening may be neither feasible nor cost-effective [20], and a more targeted approach such as symptomatic POC-LFT may be required for effective, affordable and continual control. Our study suggests that targeted symptomatic POC-LFT by clinicians in primary care followed by same-day notification and advice to isolate can effectively capture PCR-positivity (as a proxy for COVID-19 symptomatic infection) as well as COVID-19 hospitalisation trends during a major outbreak in Austria. This policy would enable early self-isolation and contact tracing of cases and hence may be a plausible alternative to indiscriminate whole population screening and can be an ongoing method for infection control in ‘living with COVID-19’ situation.

Our findings add to the evidence of POC-LFT being a good alternative to PCR testing in “living with COVID-19”. Previous results have shown that LFTs can reliably and accurately detect COVID-19 infection among symptomatic individuals [21], and when used for clinical triage in emergency department settings [22] and in primary care [3]. Moreover, a study modelling targeted symptomatic POC-LFT in outpatient settings combined with early TTI suggested that POC-LFT may be superior to PCR in curbing transmission [23]. Specifically, in [23] the authors suggested that antigen testing offered positive incremental net benefit relative to PCR in the outpatient setting and was robust to variation in most other parameter values (see Figure 4A in the technical report at http://links.lww.com/EDE/B834). They noted, however, that there was one exception of this advantage of LFT vs. PCR: if strict isolation (i.e., sufficient to reduce transmission by 70%) were maintained during the wait for PCR results, then PCR would be preferred to antigen testing in this setting.

Our results expand this evidence. Using real-life national data, here we have shown that POC-LFT testing of people presenting with flu-like symptoms during a COVID-19 outbreak in primary care, delivered at scale and combined with immediate self-quarantining and contact tracing, is feasible and accurate, and likely to exhibit its greatest benefit on dampening transmission, and reducing hospitalisations and deaths. Given the feasible scaled back of TTI under the plans for “living with COVID-19” and reduction in PCR testing, we recommend that clinical triage with POC-LFT testing of symptomatic patients and advice to self-isolate for suspected cases, would be a suitable alternative to PCR testing and large-scale TTI.

Our study has multiple strengths. Firstly, it uses data from across all 94 political districts in Austria before the COVID-19 vaccine became available in the country. During the observation period, large-scale free symptomatic POC-LFT was only available via the national policy, and LFTs were not widely accessible for home testing or at pharmacies, and this policy affected districts in different stages of epidemic growth. Using this large data set allowed for testing heterogeneity across settings to be explored and allowed evaluation of a realistic nation-wide testing policy. Secondly, most PCR results were from symptomatic testing as large-scale asymptomatic PCR screening had not been rolled out over the study period. Availability of POC-LFT reimbursement data from the largest national health insurer allowed accurate stratification of the study cohorts at district level.

Limitations of our study included the missing total number of PCR, age and gender stratification of the data for intensive care admissions and deaths, and the short observation period. This prevented us from exploring the effect of the POC-LFT testing policy on the epidemic trends in hospital occupancy and deaths from COVID-19. We found that while the number of reactive POCLFs / Total POCLFs does not differ so much in October and November; but the number of reactive POCLFs / Total Positive PCR is very different in October and November. As we didn’t have the total number of PCR tests, this was difficult to explore and address, and we will explore this in follow up studies. The short observation period may also be the reason why the trend in deaths related to COVID-19 was not aligned to the trends in symptomatic POC-LFT, PCR-positivity and hospitalisations related to COVID-19. Evidence from the first and second wave in the UK suggests that hospitalisation were the key metrics to track the pandemic status, and correlated to the reproduction number trends [24]. If in future, the vast amount of available data on the epidemic metrics is scaled back, hospitalisation data will likely remain available. Hence, considering the effect of the POC-LFT policy on the combinations of COVID-19 symptomatic case and hospitalisation trends is sufficient for this study, and for future replications of it. In addition, while the lockdown measures across the studies districts were the same, we didn’t have further data on these to include in our analysis. Should these data become available, we can extend our study in the future.

Whether high POC-LFT-activity performed in advance of a pandemic’s growth, and how this would affect such growth, we cannot answer directly from this study for a number of reasons. Firstly, we cannot distinguish between proactive and reactive POC-LFT activity; and secondly, several control variables would have been needed to explain differences in growth between regions. Hence as an alternative to this, we explored whether POC-LFT-activity is associated with COVID-19 case finding and whether high POC-LFT-activity is associated with a reduction in cases and hospitalisations in the setting of Austria’ lockdown in fall 2020.

The value of a POC-LFT strategy for future pandemics can be assessed as soon as basic information on viral dynamics becomes available. The strategy relies on the limit of detection of LFTs below or near a viral load equaling contagiousness. A technical limitation is the availability of LFTs, which may take longer to develop and produce than adapting a primer for PCR. However, given availability of an adequate LFT, the strategy should be in the toolbox for any future viral pandemic. PCR testing requires complex logistics for sample transportation and integration with healthcare IT systems for effective partner notification. Our study shows that LFT is more readily adopted by practitioners in high prevalence situations and enables immediate isolation of suspected cases, highlighting the importance of primary care in outbreak prevention and control.

In conclusion, our study demonstrates that a nation-wide policy for targeted POC-LFT of symptomatic patients attending primary care, delivered at scale, and combined with immediate isolation of LFT reactive cases and tracing of their contacts, is reflective of trends in COVID-19 cases and hospital admission. We show that such a policy can capture the epidemic increase and decline during the early stages of the second epidemic wave in Austria. Such symptomatic POC-LFT policy is an effective, and less expensive intervention for viral containment and control and should be part of any national TTI strategy.

## Supplementary Information

**Additional file 1.** Supplementary materials.

## Declarations

### Availability of data and materials

The data that support the findings of this study are available from the corresponding author but restrictions apply to the availability of these data, which were used under license for the current study, and so are not publicly available. Data are however available from the authors upon reasonable request and with permission of the corresponding author.
