# Supplementary material for: Impact of national-scale targeted point-of-care symptomatic lateral flow testing on trends in COVID-19 infections, hospitalisations and deaths during the second epidemic wave in Austria (REAP3)
Source: BMC Public Health. 2023 Mar 16;23:506. doi: 10.1186/s12889-023-15364-w (PMC10018611; doi:10.1186/s12889-023-15364-w)
Supplement: Supplementary file 1 — Additional file 1. Supplementary materials. [file 12889_2023_15364_MOESM1_ESM.docx]

# Supplementary Materials to

Impact of national-scale targeted point-of-care symptomatic Lateral Flow Testing on trends in COVID-19 infections, hospitalisations and deaths during the second epidemic wave in Austria (REAP3)

## Methods

## Data collation/synthesis

We used three different datasets for each of the 94 Austrian districts for the period between October 01 to December 31, 2020. Firstly, we used the reimbursement data of the largest Austrian health insurance provider (Österreichische Gesundheitskasse, Austrian Health Insurance Fund, https://www.gesundheitskasse.at) covering the monthly sum of reimbursed LFTs per district conducted by contracted general practitioners, and pediatric and internist practices. Secondly, we used the publicly available district-level data of people testing SARS-CoV2 PCR positive and deaths (available at https://covid19-dashboard.ages.at/). The third dataset used were daily hospital admissions for each district provided by the Austrian National Public Health Institute (Gesundheit Österreich GmbH, https://goeg.at/english). All data on PCR, hospital admissions and deaths were stratified per age group and sex.

Stratification of districts by POCLFT activity

To explore the impact of POCLFT among symptomatic patients, we stratified the 94 districts according to the number of POCLFT performed per 100,000 inhabitants between October and December 2020 into the following three population cohorts: (i) *high*, (ii) *medium* and (iii) *low*. We note that most POCLFTs (90%) were conducted by contracted general practitioners, and the remaining POCLFT (10%) were performed by contracted pediatricians and internists. The stratification was done iteratively. Firstly, we identified districts with *early* and *high* POCLFT use (number of POCLFTs per 100,000 inhabitants) in October to be among the “top third” districts regarding POCLFT activity. Instead of 31 districts (a third of 94 districts) we included 37 districts with *early* and *high* use to avoid the exclusion of districts at the margin which are similar. Districts, which remained within this “top third” until December we denoted as *high* districts. Analogously, we searched for the “bottom third” districts with the lowest POCLFT use in October, and denoted districts as *low* if POCLFT use per inhabitant remained low until December. Districts, which neither belonged to the top third nor the bottom third over the entire three-month observation period, we denoted as districts with *medium* test use.

Statistical analysis

Across the three population cohorts we undertook three separate analyses. Firstly, we determined the association between POCLFT use by cohort to the prevalence of COVID-19 related infections, hospital admissions related to COVID-19, and deaths related to COVD-19 over the three-month-period. Secondly, we quantified the differences in the slopes of COVID-19 PCR-positivity between *high* and *low* POCLFT-activity cohorts, during the rise and the decline of PCR-positivity from the (effective) beginning of reimbursed POCLFT up to the end of the national lockdown (October 25 to December 06) and using multiple group interrupted time series analysis (ITSA) (Linden & Arbor, 2015). Across 49 observations per day, 25 in the *high* and 24 in the *low* cohort, and over 42 days we used ITSA with the following assumptions a) we consider a lag for effectiveness of three days to account for the fact that the period started with the introduction of reimbursement of POCLFT by the health insurance fund to the contracted practices ; b) we divided the period as pre-peak and post-peak of infections noting that COVID-19 infections started to decline between the enforcements of the closing of the gastronomy on November 05 and the hard national lockdown on November 17; c) the period ended with the last day of the hard national lockdown on December 06 (Figure 1B).

We fitted an Ordinary-ordinary least squares (OLS) model and tested for autocorrelation of the error distribution with the actest proposed by Cumby and Huizinga.^20^ The model included two time lags and was of the following form:

$Y_{t}=\beta_{0}+\beta_{1}T_{t}+\beta_{2}{high}_{t}+ \beta_{3}{high}_{t}T_{t}+ \beta_{4}post+ \beta_{5}post{high}_{t}+\beta_{6}postT_{t} +\beta_{7}post{{high}_{t}T}_{t}+\varepsilon_{t}$ (1)

The dependent variable was the weekly rolling average of PCR positive cases (or weekly rolling average of hospital admissions) of each district. We identified the *high* district cohort with the dummy variable *high* and the time since the start of the study (October 22) with T (and t each time point). We indicated the days after the peak of COVID-19 PCR-positivity (hospital admissions) with the dummy variable *post*. The coefficients of interest were the interaction of *high* and t (*high_t*), indicating the difference in slopes before the peak, and, the interaction of post, *high* and t (*post_high_t*), indicating the difference in slopes after the peak between the cohorts with *high* and *low* LFT use.

Finally, we calculated the Pearson correlation coefficients between PCR-positivity and COVID-19 hospitalizations and between PCR-positivity and COVID -19 related deaths across the *high*, *medium*, and *low* POCLFT-activity cohorts. We accounted for lags between PCR-positivity and hospitalization due to COVID-19 and PCR-positivity and COVID-19 related deaths and determined the lag for which the Pearson correlation was strongest.

## ADDITIONAL TABLES

Table S1: Coefficients of the interrupted time series analysis of the PCR-positivity model (2^nd^ column) and hospitalizations model (3^rd^ column).

|  |  | ***PCR-positivity*** | | ***hospitalizations*** | | |  | |  |  |
| --- | --- | --- | --- | --- | --- | --- | --- | --- | --- | --- |
|  |  |  |  |  |  |  |  | |  |  |
|  | *high* | 8.5 | [0.64; 16.3] | 0.53 | [-0.26; 1.33] | |  | |  |  |
|  | *t* | 2.35*** | [2.0; 2.7] | 0.12*** | [0.07; 0.17] | |  | |  |  |
|  | ***high_t*** | 2.24*** | [1.4; 3.1] | 0.10^+^ | [0.02; 0.18] | |  | |  |  |
|  | *post* | 88.3*** | [68.4; 108.2] | 3.06** | [1.05; 5.08] | |  | |  |  |
|  | *post_high* | 52.0** | [21.9; 82.1] | 4.02** | [1.13; 6.9] | |  | |  |  |
|  | *post_t* | -4.03*** | [-4.7; -3.3] | -0.15*** | [-0.23; -0.07] | |  | |  |  |
|  | ***post_high_t*** | -3.60*** | [-4.8; -2.3] | -0.20** | [-0.32; -0.08] | |  | |  |  |
|  | *c* | 20.05*** | [15.9; 24.2] | 1.03*** | [0.47; 1.58] | |  | |  |  |
|  |  |  |  |  |  | |  | |  |  |
|  | **** p<0.001; ** p<0.005; * p<0.010; + p<0.015* | | | | |  | |  | |  |

## ADDITIONAL FIGURES

Figure S1: Fitted regression models from equation (1) as solid lines for the time series of PCR-positivity (left figure) and the time series of hospital admissions related to COVID-19 (right figure) overlaid over the data (as crosses) and plotted over the period October 25 and December 6, 2020.

|  |  |
| --- | --- |


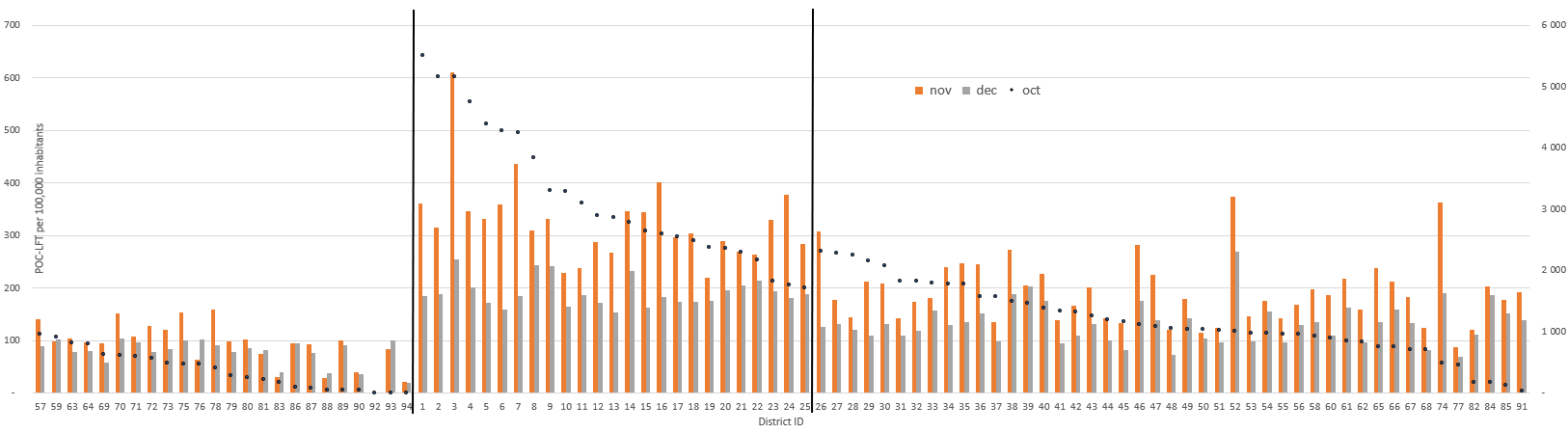


Figure S2: Stratification of 94 Austrian districts according to POC-LFT activity per 100,000 inhabitants from October to December 2020.

Note: The x-axis shows the districts’ IDs. The y-axis on the left side refers to values of October, and the y-axis on the right side refers to values of November and December. Two vertical lines separate the low, high and medium cohorts.
